# Unilateral hyperreactio luteinalis of the ovary during pregnancy mimics ovarian malignancy: A case report

**DOI:** 10.1097/MD.0000000000043590

**Published:** 2025-07-25

**Authors:** Shiyu Chen, Haiyan Yu

**Affiliations:** a Department of Ultrasonic Medicine, West China Second University Hospital of Sichuan University, Chengdu, China; b Key Laboratory of Birth Defects and Related Diseases of Women and Children (Sichuan University), Ministry of Education, Chengdu, China; c Department of Obstetrics and Gynecology, West China Second University Hospital, Sichuan University, Chengdu, China.

**Keywords:** hyperreactio luteinalis, ovarian malignancy, pregnancy, ultrasound

## Abstract

**Rationale:**

Hyperreactio luteinalis (HL) involving a unilateral ovary is extremely rare, and the clinical presentation of HL can mimic that of ovarian malignancy, resulting in potential misdiagnosis and unnecessary surgical interventions.

**Patient concerns:**

A 34-year-old female (gravida 6, para 1), pregnant for 6 weeks according to her menstrual records, presented with nausea and abdominal distension.

**Diagnoses:**

Ultrasonography revealed unilateral ovarian enlargement with multilocular cysts and ascites. Cancer antigen 125 (CA125) was elevated at 337.6 U/mL, raising suspicion of ovarian malignancy.

**Interventions:**

Surgery was performed due to the initial impression of malignancy. Intraoperative frozen section examination revealed multiple luteinized cysts, confirming HL.

**Outcomes:**

The final diagnosis of HL was established, and unnecessary radical surgical procedures were avoided. The patient has followed up for >3 years, and there are no abnormalities in her serum CA125 and gynecological ultrasound results.

**Lessons:**

HL can mimic as ovarian malignancy, when encountering pregnant women with ovarian enlargement, elevated CA125 levels, and ascites, HL should be considered as a potential differential diagnosis. This awareness is crucial for accurate diagnosis and appropriate management, helping to avoid unnecessary surgical interventions.

## 1. Introduction

Hyperreactio luteinalis (HL), a rare benign condition distinguished by bilateral ovarian enlargement and multiple cysts, is characteristically associated with multiple gestations, assisted reproductive technology, and high levels of gonadotropins.^[1]^ It is exceptionally rare to find a case of HL involving a unilateral ovary. Because the clinical presentation of HL can mimic that of ovarian malignancy, chances of misdiagnosis and unnecessary surgical interventions are very common.^[2]^

In this study, we reported a rare case of HL in a natural conception pregnancy where HL involved a unilateral ovary combined with elevated cancer antigen 125 (CA125) and ascites, and the patient’s condition was initially misdiagnosed as an ovarian malignancy. The objective of this study was 2-fold: to enhance awareness of unilateral ovarian involvement in HL by providing important information about this rare condition, and to reduce the need for nonessential surgeries based on this knowledge.

## 2. Case report

A 34-year-old female (gravida 6, para 1) was referred to our department on suspicion of ovarian cancer. The patient, who presented with nausea and abdominal distension, had been hospitalized at 6 weeks’ gestation with a regular prepregnancy menstrual cycle. Further, she had no history of hypothyroidism, ovarian induction, or polycystic ovarian syndrome. The patient had not used any drugs for ovulation induction. Clinical tests revealed abdominal distention with generalized tenderness and shifting dullness in the abdomen.

**Figure 1.**
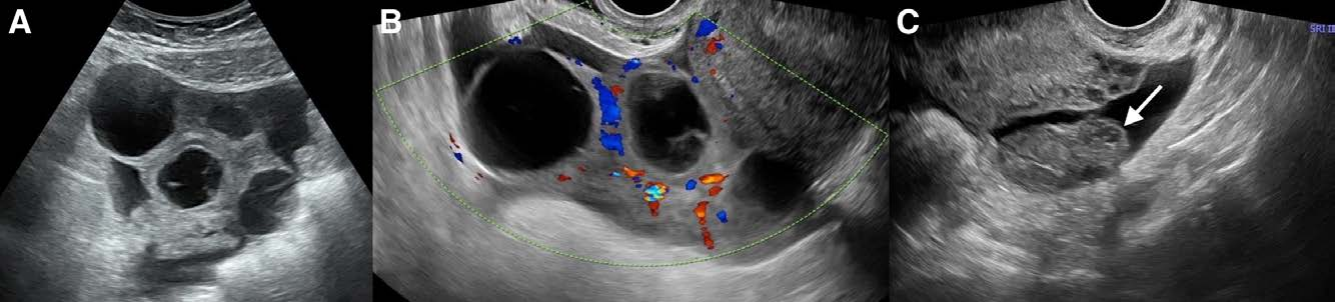
(A) Two-dimensional transabdominal ultrasound image showing enlarged right ovary with multiple thin-walled cysts. (B)Transvaginal color Doppler ultrasonography showing prominent peripheral blood flow surrounding the cyst, displaying a characteristic “spoke wheel” sign. (C) Normal-size left ovary (indicated by the arrow).

Laboratory tests showed the serum human chorionic gonadotropin (HCG) level at 4254.6 mIU/mL and the CA125 level at 337.6 U/mL (normal range: 0–35 U/mL). Transvaginal ultrasonography revealed a single, 8-mm-diameter intrauterine gestation sac with no visible yolk sac and fetal bud. The right ovary had enlarged to a size of 91 × 42 × 66 mm, with multiple thin-walled cysts, and blood flow was detected on the wall. In contrast, the left ovary was normal (Fig. 1). Free fluid was detected in the pelvic cavity, upper abdomen, and left and right paracolic gutters.

**Figure 2.**
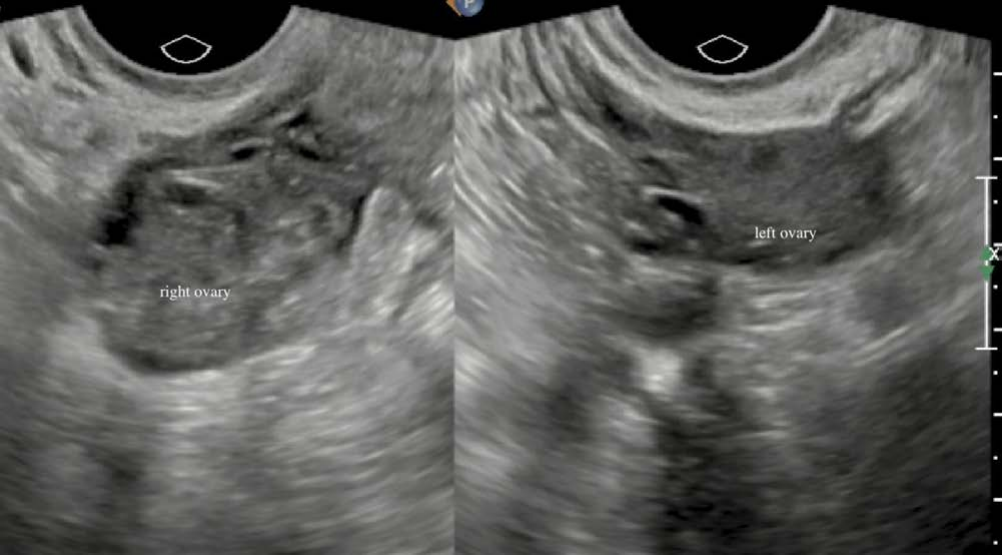
Two-dimensional transvaginal ultrasound image obtained 1 month after surgery showing regression of cysts in the right ovary. Note that both ovaries were normal in size.

Elevated tumor markers, a unilateral large ovarian mass, and ascites raised a strong suspicion of ovarian malignancy. With the patient deciding not to continue with her pregnancy, laparoscopic biopsy of the right ovarian lesion was performed along with abortion. Intraoperative frozen section examination revealed HL with multiple luteinized cysts. On the basis of this impression, the surgery was terminated, and only a biopsy was conducted without performing a total oophorectomy. Pelvic ultrasound examination performed 1 month later revealed regression of the ovarian cysts (Fig. 2). Concurrently, the serum HCG level had decreased to 13.5 mIU/mL and the CA125 level to 174.3 U/mL. To date, the patient has followed up for >3 years, and there are no abnormalities in her serum CA125 and gynecological ultrasound results.

## 3. Discussion

The relatively rare ovarian mass during pregnancy accounts for an incidence rate of 2% to 5%. Benign tumors are the most common, accounting for approximately 80% of all ovarian tumors during pregnancy.^[3]^ Some ovarian masses are functional and disappear after delivery.

HL is a rare luteal phase dysfunction whose pathogenesis is not fully understood. According to some studies, HL may be caused by the dual stimulation of progesterone and HCG, resulting in the proliferation and cystic dilation of luteal tissue.^[4]^ HL usually resolves spontaneously in the later stages of pregnancy. Since HL is a self-limiting condition, conservative management is the primary treatment. Surgery may be necessary in cases of complications such as torsion, rupture, or suspicion of malignancy.^[1]^

In our case, misdiagnosis as an ovarian malignancy primarily resulted from the presence of a large, multilocular cyst in the unilateral ovary, in addition to ascites and the elevated tumor marker CA125, which is generally used as a tumor marker in the diagnosis of ovarian cancer. However, its specificity is limited because CA125 concentrations can be affected by pathophysiological conditions such as the menstrual cycle and pregnancy, as well as some benign gynecological conditions, including endometriosis and pelvic inflammatory disease.^[5]^ Reportedly, in 9% to 35% of normal pregnant women, serum CA125 levels are higher than the cutoff value of 35 U/mL, and the levels are at their highest in the first trimester, with the maximum value reaching 550 U/mL.^[6,7]^ Elevated CA125 levels have also been reported in pregnant women with HL, and the highest reported level in the literature is 442 U/mL.^[8]^ Given the effect of pregnancy on maternal serum CA125 concentration, the diagnostic value of elevated CA125 levels for ovarian masses during pregnancy remains controversial.

When an adnexal mass is detected during pregnancy, it becomes essential to differentiate between benign conditions and malignancy. Ultrasonography is the primary method of examination for adnexal masses during pregnancy. The ultrasound features of HL are multiple thin-walled luteal cysts with a characteristic “spoke wheel” sign, while the features of malignant tumors include the presence of ascites, irregular solid components, at least 4 papillary structures, multilocular cystic-solid tumors with a diameter >100 mm, and increased vascularity on color Doppler examination.^[1,9]^ Magnetic resonance imaging is highly valuable in evaluating adnexal masses, especially when ultrasound findings are inconclusive. With superior soft tissue contrast and high specificity, it helps differentiate benign from malignant lesions and provides additional diagnostic clarity to guide clinical decision-making.^[10]^

Most HL cases lead to bilateral ovary enlargement with multiple cysts. Cavoretto et al,^[2]^ who reviewed the HL literature, reported a case from their group. They found that bilateral ovary enlargement with multiple follicular cysts was common across all of the 65 cases they had studied. Surgical intervention was found to be more common when the HL diagnosis was difficult. In the study by Cavoretto et al,^[2]^ surgery was performed in 47% of the reported HL cases, 38% of which were suspected cases of malignancy. Nevertheless, only 1 case of HL causing unilateral ovary enlargement has been reported so far. A study by Watkins et al^[4]^ documented 10 cases of HL during pregnancy. Only 1 of these cases involved the incidental discovery of unilateral ovary enlargement during a cesarean section at 36 weeks of a singleton pregnancy, subsequently leading to bilateral subtotal oophorectomy. In both the previously reported case and our own, surgical intervention was pursued on suspicion of malignancy. The lack of knowledge about HL cases where a single ovary is affected may have played a major role in the decision to initiate surgical treatments.

In conclusion, our case highlights the importance of clinical awareness and appropriate diagnostic evaluation in distinguishing HL from ovarian malignancy, specifically when the condition is limited to a single ovary. An early and accurate diagnosis of this benign condition can ensure optimal management while reducing unnecessary surgical interventions.

## Acknowledgments

We thank LetPub (www.letpub.com) for its linguistic assistance during the preparation of this manuscript.

## Author contributions

**Project administration:** Shiyu Chen.

**Writing – original draft:** Shiyu Chen.

**Supervision:** Haiyan Yu.

**Writing – review & editing:** Haiyan Yu.
